# PIXER: an automated particle-selection method based on segmentation using a deep neural network

**DOI:** 10.1186/s12859-019-2614-y

**Published:** 2019-01-18

**Authors:** Jingrong Zhang, Zihao Wang, Yu Chen, Renmin Han, Zhiyong Liu, Fei Sun, Fa Zhang

**Affiliations:** 1 High Performance Computer Research Center, Institute of Computing Technology Chinese Academy of Sciences, No. 6 Kexueyuan South Road, Haidian District, Beijing, 100190 China; 2 University of Chinese Academy of Sciences, Beijing, China; 3 Computer, Electrical and Mathematical Sciences and Engineering (CEMSE) Division, King Abdullah University of Science and Technology (KAUST), Computational Bioscience Research Center (CBRC), Thuwal, 23955-6900 Saudi Arabia; 4 National Laboratory of Biomacromolecules, CAS Center for Excellence in Biomacromolecules, Institute of Biophysics, Chinese Academy of Sciences, 15 Datun Road, Beijing, 100101 China; 5 Center for Biological Imaging, Institute of Biophysics, Chinese Academy of Sciences, 15 Datun Road, Beijing, 100101 China

**Keywords:** Cryo-electron microscope, Single-particle analysis, Deep learning, Particle selection, Segmentation

## Abstract

**Background:**

Cryo-electron microscopy (cryo-EM) has become a widely used tool for determining the structures of proteins and macromolecular complexes. To acquire the input for single-particle cryo-EM reconstruction, researchers must select hundreds of thousands of particles from micrographs. As the signal-to-noise ratio (SNR) of micrographs is extremely low, the performance of automated particle-selection methods is still unable to meet research requirements. To free researchers from this laborious work and to acquire a large number of high-quality particles, we propose an automated particle-selection method (PIXER) based on the idea of segmentation using a deep neural network.

**Results:**

First, to accommodate low-SNR conditions, we convert micrographs into probability density maps using a segmentation network. These probability density maps indicate the likelihood that each pixel of a micrograph is part of a particle instead of just background noise. Particles selected from density maps have a more robust signal than do those directly selected from the original noisy micrographs. Second, at present, there is no segmentation-training dataset for cryo-EM. To enable our plan, we present an automated method to generate a training dataset for segmentation using real-world data. Third, we propose a grid-based, local-maximum method to locate the particles from the probability density maps. We tested our method on simulated and real-world experimental datasets and compared PIXER with the mainstream methods RELION, DeepEM and DeepPicker to demonstrate its performance. The results indicate that, as a fully automated method, PIXER can acquire results as good as the semi-automated methods RELION and DeepEM.

**Conclusion:**

To our knowledge, our work is the first to address the particle-selection problem using the segmentation network concept. As a fully automated particle-selection method, PIXER can free researchers from laborious particle-selection work. Based on the results of experiments, PIXER can acquire accurate results under low-SNR conditions within minutes.

## Background

Single-particle cryo-electron microscopy (cryo-EM), which acquires the three-dimensional (3D) structures of protein and macromolecular complexes from two-dimensional (2D) micrographs, is gaining popularity in structural biology [1]. Many high-resolution structures have been reported [2, 3]. These high-resolution results typically rely on hundreds of thousands of high-quality particle images selected from the micrographs.

However, particle selection still presents many challenges. One troubling feature is the low signal-to-noise ratio (SNR) of micrographs. As high-energy electrons can greatly damage the specimen during imaging, their dose must be strictly limited, which results in extremely noisy micrographs. Further, much interference arises from sources such as ice contamination, background noise, amorphous carbon and particle overlap. High-resolution reconstruction requires extensive particles identification. For example, to acquire the cryo-EM structure of the activated GLP-1 receptor in a complex with a G protein, researchers used 620,626 particles [2]. The massive demand for particles further intensifies the challenges of particle selection. In a realistic experimental procedure, researchers spend days to weeks manually or semi-automatically selecting particles, which is a laborious, time-consuming and frustrating process.

Over the past decades, many different automated or semiautomated particle-selection methods have been proposed. There have been many particle-selection tools such as Picker [4], RELION [5] and XMIPP [6], most of which are based on techniques adopted from conventional computational vision, such as edge detection, feature extraction, and template matching. However, these methods are not suitable for micrographs with poor contrast and low SNR, as their performance declines significantly with decreasing micrograph quality.

During the past few years, deep learning has grown progressively. By using features from big data analyses and generating layered features from deep neural networks, deep learning can outperform many conventional techniques in computational vision [7]. Furthermore, some deep learning applications have shown robustness against low SNRs [8]. As the size of cryo-EM data continually increases while the SNR of micrographs remains low, deep learning appears to be well suited for processing cryo-EM data. To date, three methods have been proposed to select particles based on deep learning, namely, DeepPicker [9], DeepEM [10] and FastParticlePicker [11]. DeepEM still requires hundreds of particles to be manually selected by humans for training data. DeepPicker converts particle picking to an image classification problem; it crops micrographs with a sliding window and classifies these subimages into particles or background. Considering the absence of training data, DeepPicker uses other molecules as training data to train the network. FastParticlePicker is based on the object-detection network Fast R-CNN [12], which comprises a ‘region-of-interest proposal’ network and a classification network. However, instead of proposing regions of interest for micrographs, FastParticlePicker crops micrographs with a sliding window; therefore, its performance mainly relies on the classification network. As the major components of the FastParticlePicker and DeepPicker methods are similar, we choose to compare our method with in experiments.

These three methods have brought significant contributions to the particle-selection problem. However, they all overlook three common issues. First, there is no sufficient and diversified training dataset. As mentioned, the training dataset is hard to acquire. Previous work has used two to four different kinds of particles as a training dataset. However, this insufficient and undiversified dataset easily produces biased features and results in overfitting of some features. Without a sufficient training dataset, the method cannot take advantage of the network for accommodating noisy data. Second, the current methods are based on a sliding window, which may generate a considerable number of false-positive (FP) images that waste time and memory. Third, there has not been enough attention paid to the issue of accommodating low-SNR images. Existing methods may suffer a significant performance reduction when the SNR is low.

To address these three challenges, we propose an automated particle-selection method. First, to accommodate low-SNR conditions, we designed a segmentation network to convert the noisy micrographs to probability density maps [13]. The probability indicates the likelihood of one pixel belonging to a particle. As the probability value is determined by the surrounding information, particle selection from probability density maps can produce more robust signals than direct selection from original noisy micrographs. Our work is the first to solve the particle-selection problem using segmentation networks. As segmentation is also known as ‘pixel-wise classification’, we combined the word ‘pixel’ with ‘picker’ to name our method ‘PIXER’. Further, there is currently no training dataset for particle segmentation in cryo-EM. To implement our idea, we developed an automated method to generate a training dataset for segmentation. Additionally, to enrich the diversity of our training dataset, we adopted both real cryo-EM micrographs and simulated data. Finally, we developed a grid-based, local-maximum method to acquire particle coordinates from the probability density maps. In our experiments, we used simulated and real-world datasets to evaluate performance. The results indicate that, as a fully automated method, PIXER can acquire results as good as the semi-automated methods RELION and DeepEM.

## Methods

**Fig. 1:**
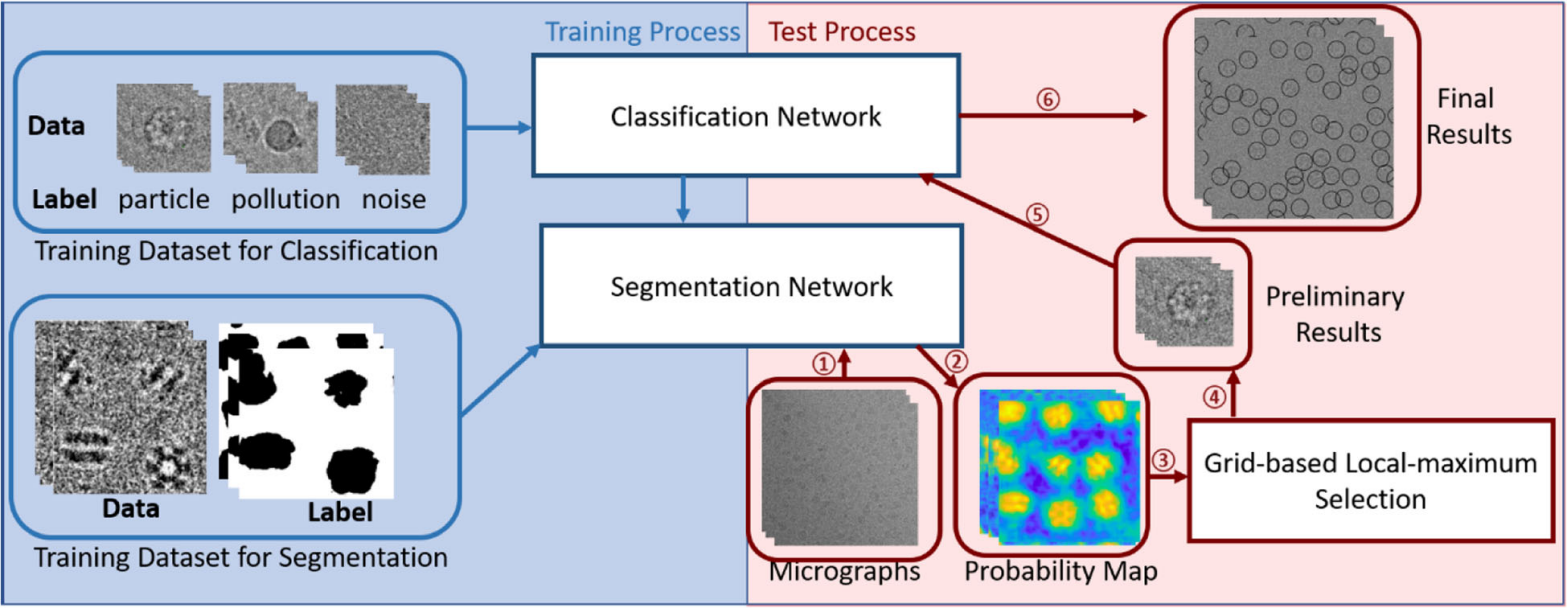
The general workflow of the training and test processes of PIXER. The blue part of the image shows the training process for segmentation and classification network. The red part of the image shows the general flow of the test process. The test process works as follows: ①feed micrographs into the segmentation network; ② acquire probability density maps from the network; ③feed density maps to a selection algorithm; ④ generate the preliminary particle coordinates from probability density maps; ⑤ feed the preliminary results into the classification network; and ⑥ generate the results after removing false positive particles

As our method is based on deep learning, we had to consider two separate aspects: the training process and the test process. The training process aims to train the networks (shown in the left part of Fig. 1). As our segmentation network is based on a classification network, we first trained the classification network and then used its parameters as initial values for the segmentation network to accelerate its training process. In this section, we first introduce our network design and the method for preparing the training dataset to complete the training process.

Here, the test process refers to the procedure of generating particle coordinates with the trained network (shown on the right side of Fig. 1). The test process has three steps: 1. feed micrographs into the segmentation network and acquire probability density maps from the network (①② in Fig. 1); 2. generate the preliminary particle coordinates from probability density maps using grid-based local-maximum method (③④ in Fig. 1); 3. feed the preliminary results into the classification network to remove FP particles (⑤⑥ in Fig. 1).

### Design of the Network

Existing networks for particle selection are based on classification networks with 3 to 5 convolution layers [9]. To support additional features and diversity, we used additional layers and channels in our classification network. In general, two networks are proposed in our method: segmentation and classification, the former of which will be first introduced as it is the cornerstone of the later.

**Fig. 2:**
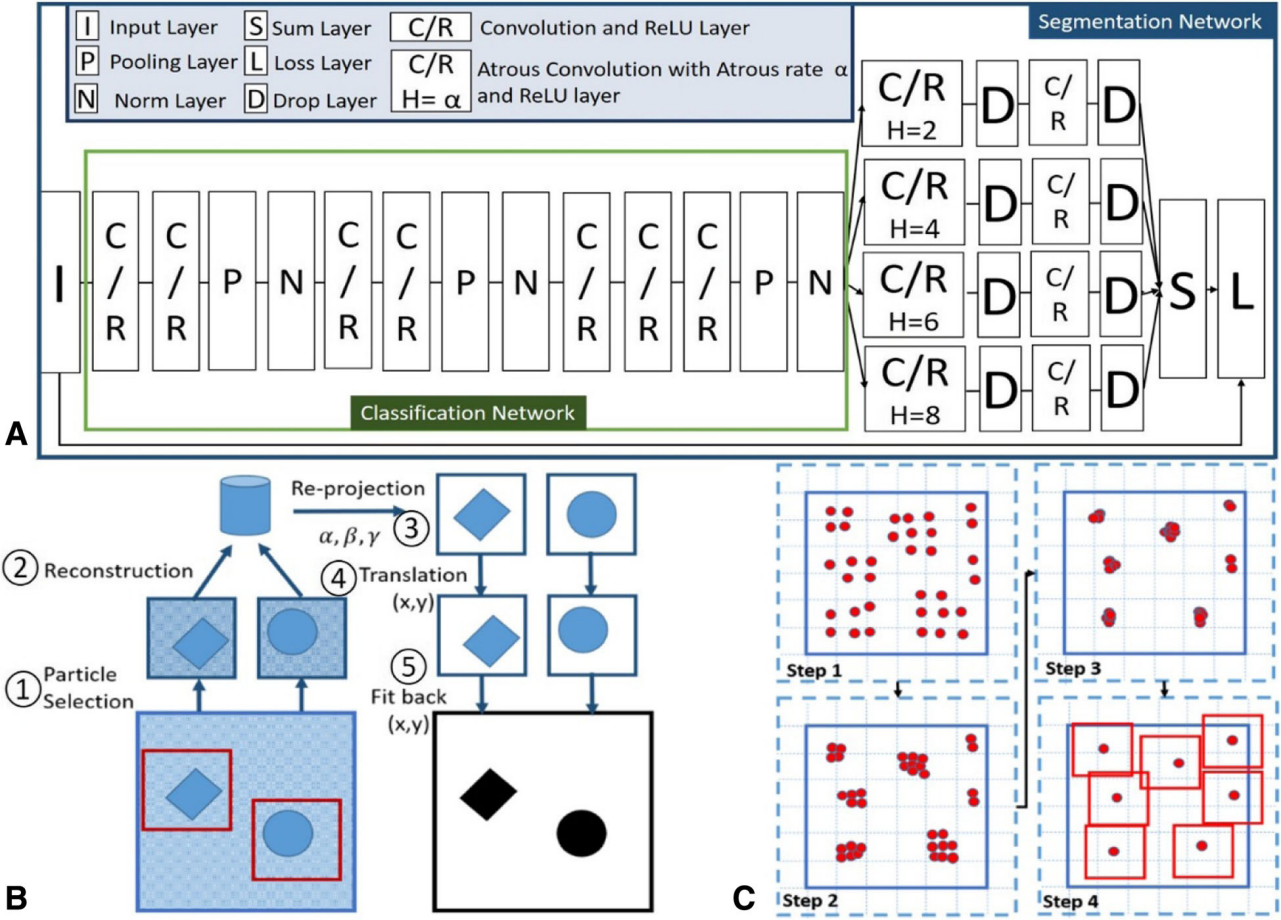
Illustrations of the PIXER methods. (**a**) The architecture of the classification and segmentation networks. (**b**) Workflow of generating training data for segmentation. ① Select particles from micrographs. The coordinates can come from manual or semi-manual particle selection software. ② Perform reconstruction using mainstream software, such as RELION and EMAN. Record the fine-tuned Euler angles and translation parameters. ③ Generate corresponding re-projection images for each particle. ④ Adjust the coordinates based on the translation parameters. ⑤ Fit these re-projection images back into the label image of each micrograph. (**c**) Procedure for the grid-based, local-maximum particle-selection method. Step 1: Generate the maximum value for each grid. Steps 2 and 3: Perform a parallel local-maximum searching method to locate local-maximum values during the iteration. Step 4: Select the local-maximum results

Fig. 2a shows the architecture of our network. The green rectangle marks the main part of the classification network. In this figure, ‘C/R’ indicates a convolution layer and a ReLU layer

Convolutional layers apply a convolution operation to the input, passing the result to the next layer. Its concrete formula can be expressed as Formula 1. In Formula 1, X indicates the input of convolutional layer. In our network, X is three dimensional, whose first dimension indicates the index of its channels. *X*_*m*, *i*, *j*_ is the point in X at coordinate (*i*, *j*) in channel *m*. In Formula 1, X owns ‘M’ channels, and *Y* indicates its output. Formula 1 calculates the value of Y at point (*i*, *j*) using convolution kernel *W* with size *M* ∗ *K* ∗ *K*.

1 $$ {Y}_{i,j}=\sum \limits_{m=0}^{M-1}\sum \limits_{k=0}^{K-1}\sum \limits_{l=0}^{K-1}{W}_{mkl}^n{X}_{m,k+i-1,l+j-1} $$

ReLU layer is the most commonly used activation function in deep learning models. The function returns 0 if it receives any negative input, but for any positive value X, it returns that value back (*ReLU*(*X*) = max(0, *X*)). ‘N’ is a ‘Norm’ layer to perform local response normalization, which normalize the input data *X*_*i*_ (*i* is the index of channel) with values from nearby channels ${X}_{i-\frac{I}{2}}$ to${X}_{i+\frac{I}{2}}$. Each value of *X*_*i*_ is divided by ${\left(1+a\sum \limits_{i=0}^I{X}_i^2\right)}^b$, where *a* and *b* are the scaling parameter and exponent parameter with default value 10^−4^ and 0.75, respectively. ‘P’ stands for the pooling layer. Inspired by previous classification network, we adopt max pooling layer (max(*X*_*k* + *i* − 1, *l* + *j* − 1_) *k*, *l* ∈ [0, *L* − 1]) in our network to resize the data layer. L is the size of sub-regions to be downsampled by max pooling.

Further, ‘I’, ‘D’, ‘S’ and ‘L’ indicate ‘Input’, ‘Drop’, ‘Sum’ and ‘Loss’ layers, respectively. The classification network takes both particle and non-particle images as inputs. Then it outputs the probabilities of the input being a particle. For the purpose of simplicity, the fully connected layer and loss layer of the classification network, which are common in other classification networks, are not depicted in Fig. 2a [9].

As shown, the segmentation network is based on the classification network. The parameters of the classification network are used as the initial values for the segmentation network to reduce the training time and increase the accuracy of the segmentation network.

The particle size in different datasets can vary from 100 × 100 to 800 × 800. To enable our network to process particles of multiscale datasets, we added the ‘Atrous convolution’ feature from ‘Deeplab’ [14] into our segmentation network. Different from traditional convolution, Atrous convolution uses filters ‘with holes’ to sample the images [14]. In Atrous convolution, we use the parameter ‘Atrous rate’ (*s*) to define the sampling rate. When Atrous rate *s* = 1, the Atrous convolution kernel is the standard convolution. For *s* > 1, Atrous convolution demenstrates down-sampling effect. Taking a 3*3 Atrous kernel with Atrous rate *s* = 2 as example, it will have the same field of view as a 5 × 5 traditional kernel, while only using 9 parameters (the rest parameters are zero). One major benefit of Atrous convolution is that it can deliver a wider field of view with fewer parameters at low computational cost. Additionally, with different Atrous rate, the same kernel parameter can process object at different scales.

In addition, multiple parallel Atrous convolution channels with different sampling rates ensure the processing of multiscale particles. We adopted four different kinds of Atrous rates (*h* = [2, 4, 6, 8]). By replacing the classical fully connected layers in the classification network with multiple parallel Atrous convolution channels, we converted the classification network to a segmentation network.

### Automated method to generate the training dataset for segmentation

The quality of the training dataset plays a significant role in the performance of the training network. However, in single-particle analysis, there is no training dataset for segmentation, and manual labeling of micrographs by humans cannot be trusted due to the extremely low SNR of images. Because many researchers have uploaded their results and initial or intermediate data to EMDataBank [15] and EMPIAR [16], we developed an automated method to generate segmentation-training datasets using these real-world datasets. For these datasets, their coordinates have already been generated from other particle selection methods and examined by researchers. So, the non-particles in micrographs are eliminated. Figure 2b shows the procedure. First, we extracted particles from each micrograph and used these particles to reconstruct the structure. During the reconstruction procedure, the translation and Euler angle parameters of each particle image were tuned. After the reconstruction, we considered the high-resolution reconstruction result as the ground truth to generate the reprojected images with corresponding Euler angles. Then, the reprojected images were adjusted according to the translation parameters to fit the selected particles. As the reprojection background has a high SNR, binarization of the reprojections represents the segmentation results of the corresponding particle images. Finally, we acquired the micrograph segmentation results using the coordinates of particles and their segmentation results.

As mentioned, reprojections of high-resolution results are more reliable than human eyes. Furthermore, much research has revealed that deep learning is robust and greatly reduces noise [17]. The results in later experiments show that the training dataset generated by this method is qualified to train the network. Using this method, we generated a sufficient and diversified dataset to train the segmentation network. For the first time, a segmentation network was applied to the particle-selection task in cryo-EM.

We also generated simulated projection images from hundreds of different kinds of particles from the EMDataBank using the simulation software InSilicoTEM [18]. To enrich the training and test dataset, the parameters (such as electron dose and pixel size) are essentially selected from a certain range randomly. The last column of Table 1 shows the ranges of these parameters.

**Table 1:** Data used in the training datasets

Name	10,017	10,028	10,081	10,097	GroEl	SIMU
Electron Dose (e/Å**2)	24.0	20.0	1.26	82.0	30	[20,50.0]
Nominal CS (mm)	2.00	2.00	–	2.70	–	[2, 3]
Defocus Max (nm)	4962	3800	3300	3500	2400	[2200,3700]
Defocus Min (nm)	1359	800	1500	1000	1000	[800,1500]
Symmetry	D2	C1	C4	C3	D7	–
Number of Images	84	90	124	153	25	496
Particle Size	177	360	256	256	140	[100, 256]
Number of Particles	42,468	13,942	16,666	51,844	6121	18,746
Pixel Size (Å)	1.77	1.34	1.3	1.31	1.3	[1.3,1.8]
Size of Micrograph	4096*4096	4096*4096	3710*3838	3838*3710	3838*3710	1024*1024

In addition, as the translation and Euler angle of each particle image can be generated by mainstream software, such as RELION and EMAN, we can apply this automated method to generate an incremental training dataset and incrementally optimize the model.

### Grid-based, local-maximum particle-selection method

The segmentation network takes micrographs as inputs and outputs the corresponding probability density maps. However, we are still one step away from our final goal: determining the coordinates of particles. In this section, we introduce the method for generating particle coordinates from the probability density maps.

First, we converted each pixel in the density map to the score of the candidate particle centered on it. For the candidate particle (centered at coordinate (m,n)) with particle size *s* × *s*, the score of the candidate is $score\left(x,y\right)=\sum \limits_{x=-\frac{s}{2}}^{\frac{s}{2}}\sum \limits_{y=-\frac{s}{2}}^{\frac{s}{2}}{W}_{x,y}{V}_{m+x,n+y}$, where *V*_*m*, *n*_ is the value of pixel at density map (m,n). *W*_*x*, *y*_ is a Gaussuan kernel of size *s* × *s*, which gives more influence on the center pixels. One benefit of using *W*_*x*, *y*_ is that when particles are close to each other, we can reduce the interference from other particles and locate the particles more precisely.

As mentioned, overlapped particles should not be selected. Therefore, we divided the micrograph into small grids and generated only one maximum candidate from each grid (shown in Step 1 of Fig. 2c). As we know, when particles are overlapped, we always choose at most one from them. Therefore, the grid size is chosen based on the particle size. For a dataset with particle size *s* ∗ *s*, the grid size will be set to $\frac{s}{2}\ast \frac{s}{2}$ in our experiment, so that the maximum overlapping area of selected particles will not exceed $\frac{s^2}{4}$. Using a micrograph 4096 × 4096 in size as an example, the number of candidates is 16,777,216, which is too high for subsequent processing. However, with a grid size of 100 × 100, the number of candidates is 41 × 41 = 1681. Next, we performed a parallel local-maximum searching method to calculate the particle coordinates. Each thread covers one candidate. As shown in Step 2 and Step 3 of Fig. 2c, in each iteration, the candidate is moved to the new maximum value in the searching area. Gradually, the threads converge to some local maximum after several iterations. As the number of candidates is limited and this step is conducted with a GPU, this procedure is completed within seconds.

At this point, the preliminary results from the probability density map can be generated. However, as we mentioned, there are many interference factors in the micrograph, and we already have a classification network that can distinguish interference factors from particles. Before obtaining the final results, therefore, we feed the preliminary results into our classification network to reevaluate the data and remove FP particles.

## Results and discussion

In this section, we first list the information for the training datasets. Then, we evaluate the performance of the segmentation network and show examples of its outputs. Selected results of the grid-based, local-maximum method are shown. To test the performance of PIXER, we tested the method on simulated and real-world datasets and compared the results with those of RELION, DeepEM and DeepPicker. After that, we show the computational efficiency.

### Training datasets

The training datasets for classification and segmentation were both composed of real-world and simulated data. For the real-world data, five different datasets were used to build the training dataset: beta-galactosidase (EMPIAR10017 [19]), *Plasmodium falciparum* 80S ribosome (EMPIAR10028 [20]), cyclic nucleotide-gated ion channel (EMPIAR10081 [21]), influenza hemagglutinin trimer (EMPIAR10097 [22]) and GroEl [23]. Additionally, we used 321 different kinds of structures to generate the simulated data. The information related to these data is listed in Table 1. The parameters of InsilicoTEM is essentially randomly selected from the ranges shown in the last column of Table 1. For the classification training dataset, we selected 5000 particles from each dataset. For the segmentation-training dataset, we randomly extracted 10,000 micrographs with sizes of 512 × 512 from each of the datasets. As shown in Table 1, we used different kinds of structures to enhance the diversity of the training dataset.

**Fig. 3:**
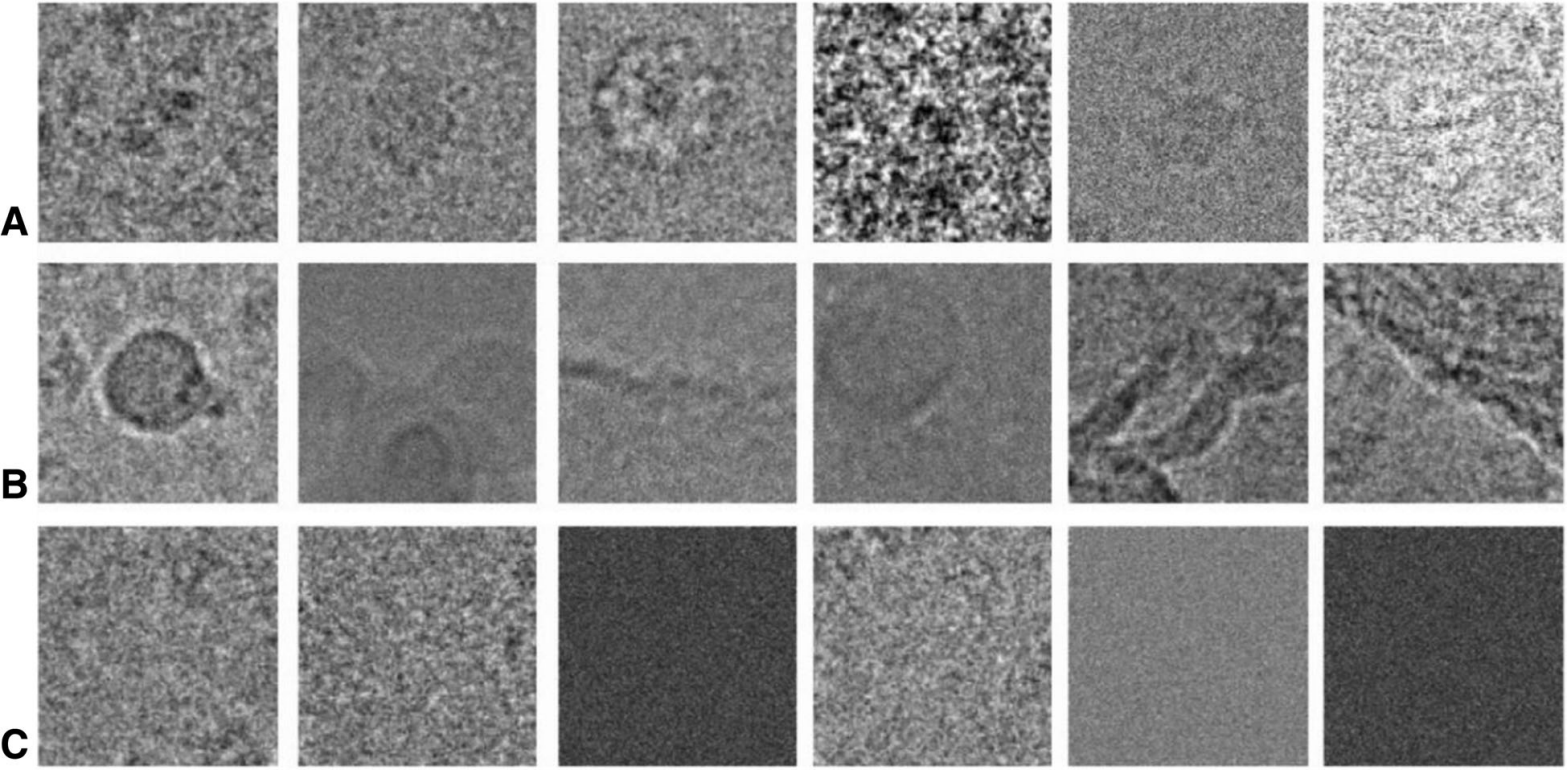
Examples of three different kinds of visual features. (**a**) Examples of particles. (**b**) Examples of interference factors. (**c**) Examples of noise images

The classification network is a 3-way network. In addition to the particle images, we processed 30,000 ice contamination images and noise background images. In Fig. 3, we illustrate examples of these three different kinds of particles. The structures of the particles differ greatly, and the SNR is relatively low.

**Fig. 4:**
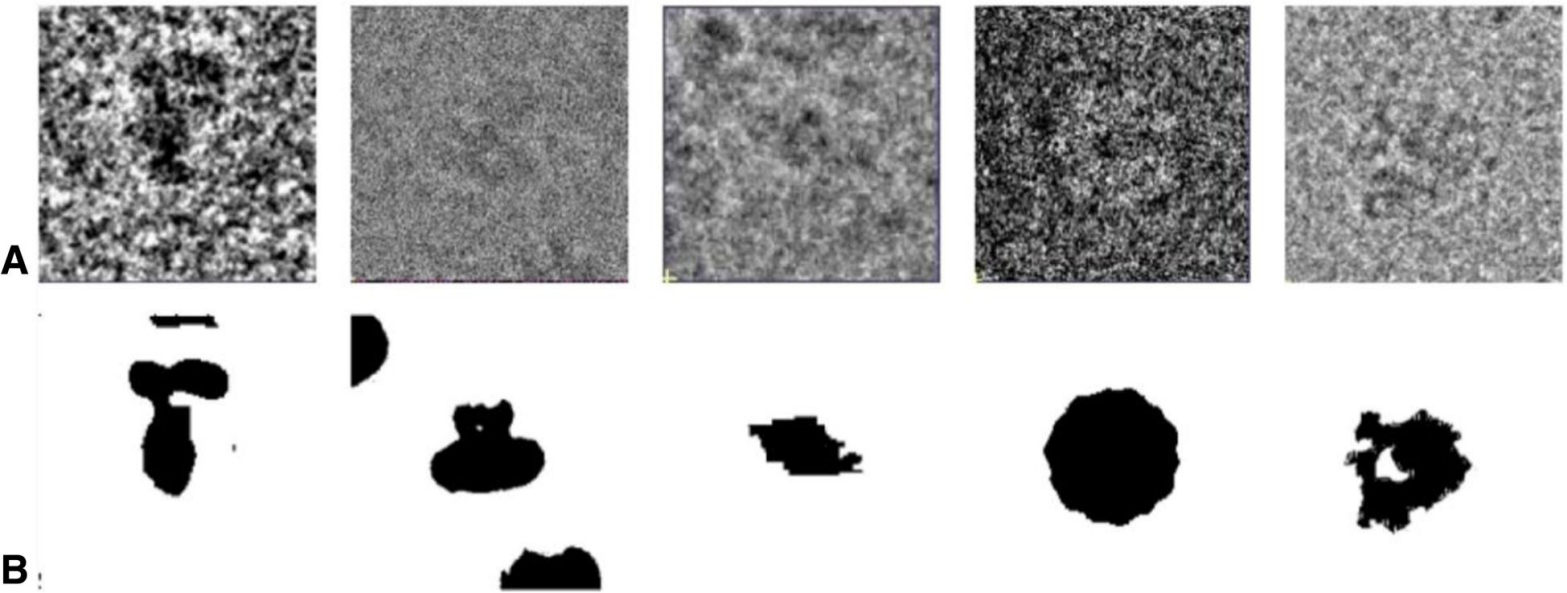
Examples of the training data for segmentation. (**a**) Examples of particles. (**b**) Corresponding segmentation results

For the segmentation-training dataset, we listed examples of the segmentation results for each particle in Fig. 4. The first column of Fig. 4 shows the simulated data. The segmentation results of simulated data were generated from the noise-free projection. The remaining images represent the segmentation results of real-world datasets. The precision of the segmentation results is assured by the high resolution of our results.

One thing needs to be clarified is that our particle selection method can be used as full-automatic particle selector. The model trained by these 5 real-world datasets and hundreds of simulated datasets can be used directly for any kinds of new datasets. The following results is acquired based on these training datasets. Meanwhile, as we developed an automated method to generate training dataset for segmentation, new datasets can be used to refine our model easily.

### Performance of the segmentation network

To test the performance of the segmentation network, we selected 5000 micrographs of size 512 × 512 as a validation dataset in addition to the training dataset. We trained five different kinds of segmentation networks with 1 to 5 Atrous convolution parallel channels. We used the pixel intersection-over-union (IOU) criteria to evaluate their performance [27] as follows:

2 $$ IOU=\frac{GroundTruth\cap Segmentation\ Result\ }{GroundTruth\cup Segmentation\ Result} $$

**Fig. 5:**
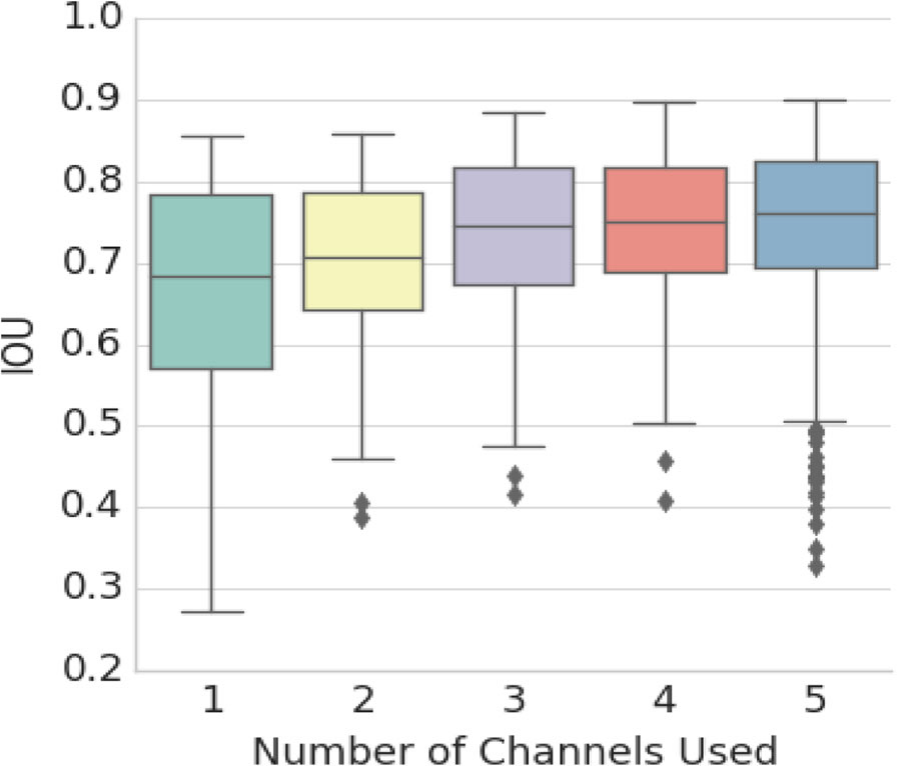
Performance of the 5 segmentation networks. To choose the appropriate number of parallel Atrous channels for the segmentation network, we trained five different networks separately. The number of parallel Atrous channels these networks are 1 to 5, respectively. In order to control variables, the training dataset, initial parameters from the classification network and all the meta-parameters (except the number of parallel Atrous channels) of these five networks are the same. We test the performance of the five segmentation networks with 5000 randomly selected micrographs 512*512 pixels in size from the data shown in Table 1 to form a validation dataset. We used intersection-over-union ($IOU=\frac{GroundTruth\cap Segmentation\ Result\ }{GroundTruth\cup Segmentation\ Result}$) statistical results to judge the performance

The box plot in Fig. 5 shows the statistical information of the IOU values for these five networks. The average performance of these networks improves, and the variance of the results declines as the number of Atrous convolution channels increases. These results show that additional Atrous convolution layers tend to stabilize the results. Additionally, we found that the performances of four and five Atrous convolution layers are essentially equal. Considering the required memory and time for training and testing networks, we chose to use four parallel Atrous convolution channels in our network.

### Examples of outputs of the segmentation network

**Fig. 6:**
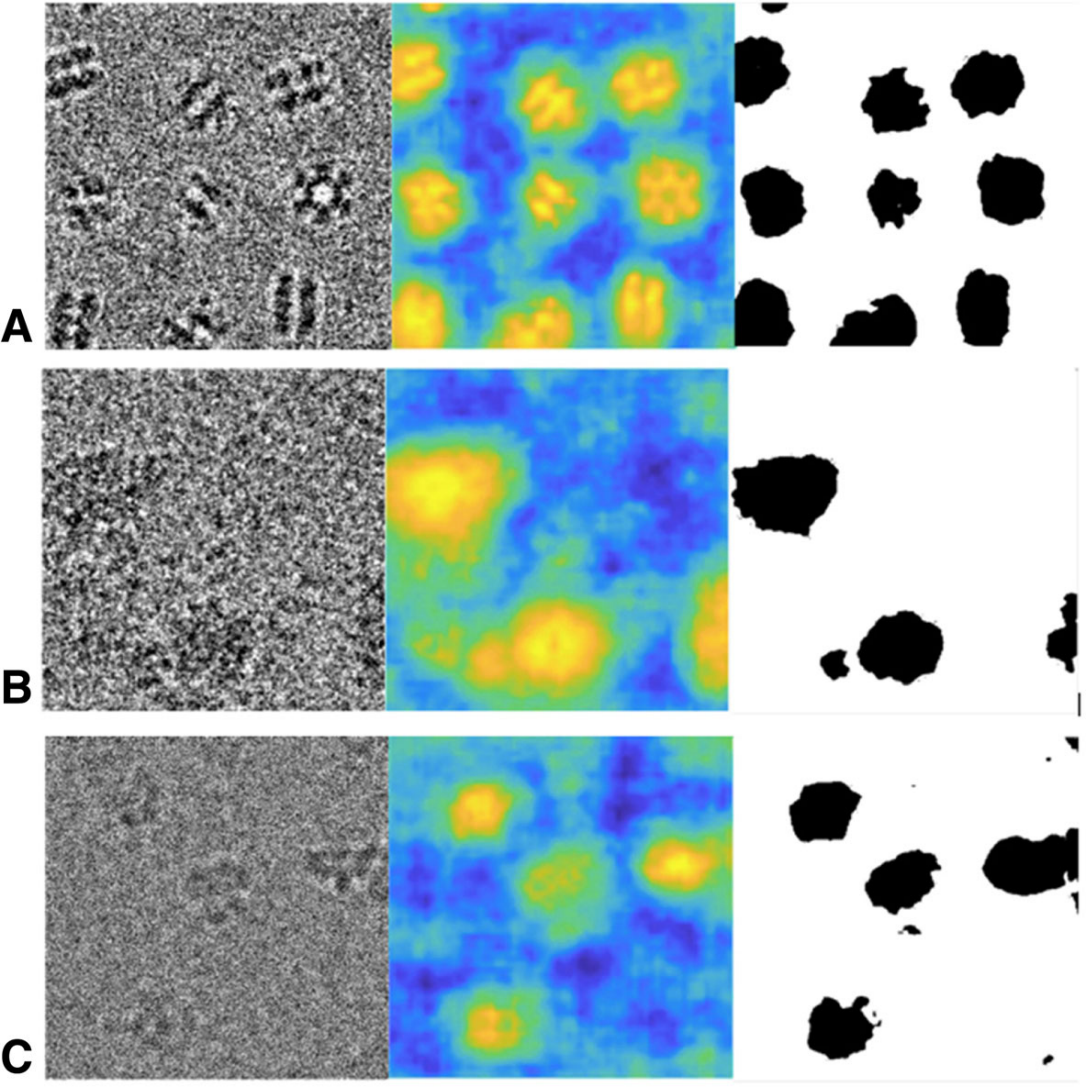
Examples of the segmentation results. (**a**) Examples from GroEL. (**b**) Examples from EMPAIR-10028. (**c**) Examples from EMPIAR-10081

We visualize the segmentation results in Fig. 6. The original micrographs, their probability density maps, and the corresponding binarized segmentation results are shown in Fig. 6. These micrographs were derived from the validation dataset mentioned above. The density map intuitively shows that even for micrographs with extremely low SNR, our segmentation network generates a dense map for locating the position of particles.

### Illustrations of the grid-based, local-maximum method

**Fig. 7:**
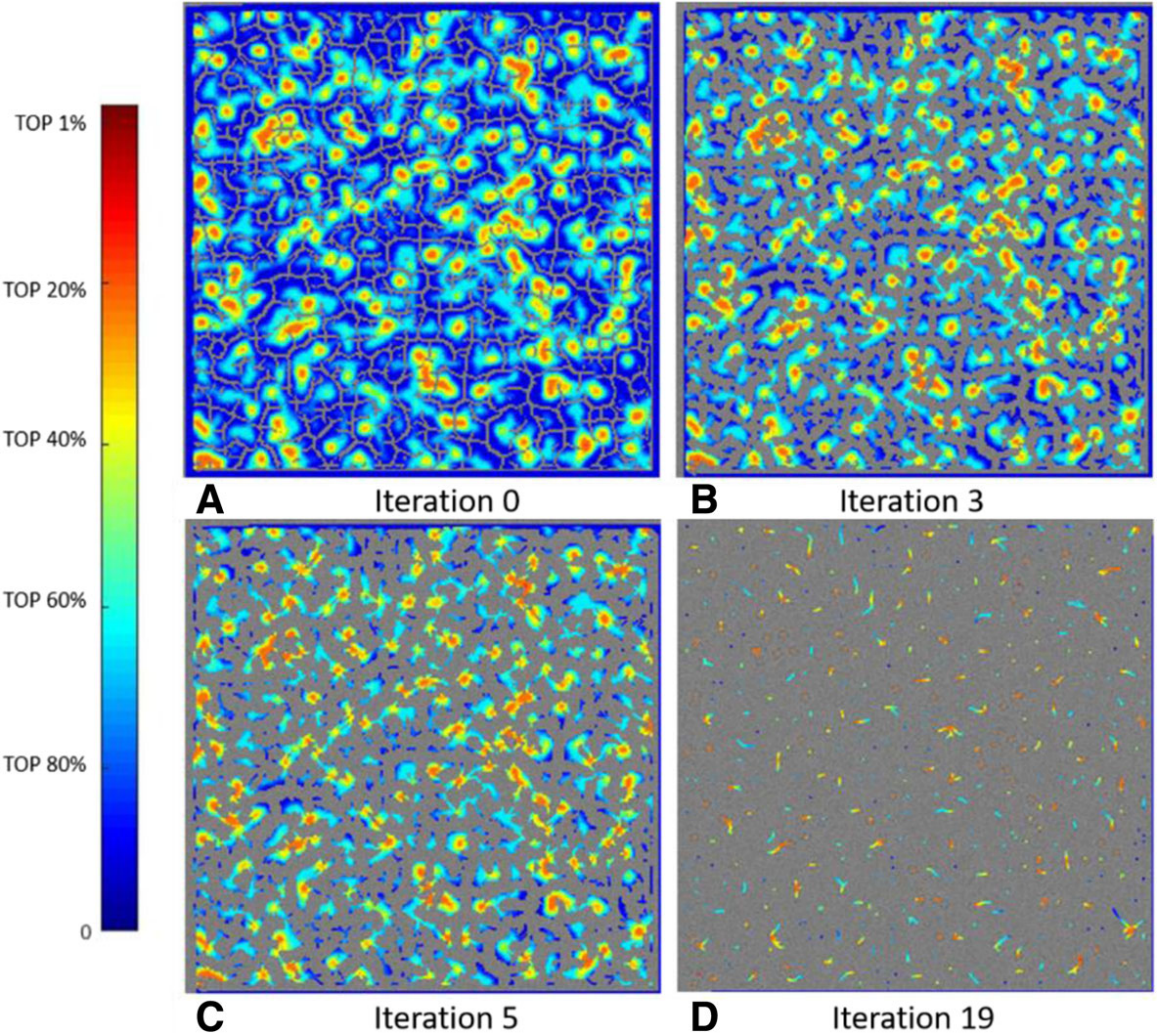
Four representative intermediate results of the grid-based, local-maximum method using one whole micrograph from dataset TRPV (EMPIAR-10005)

To select particles from the heat map, we applied a grid-based, local-maximum method. Here, we list selected intermediate results during the iterations. To show the process more clearly, we use a small grid size. Each colored point in Fig. 7 indicates a local maximum value, and the color is determined by the score of the corresponding particle.

**Fig. 8:**
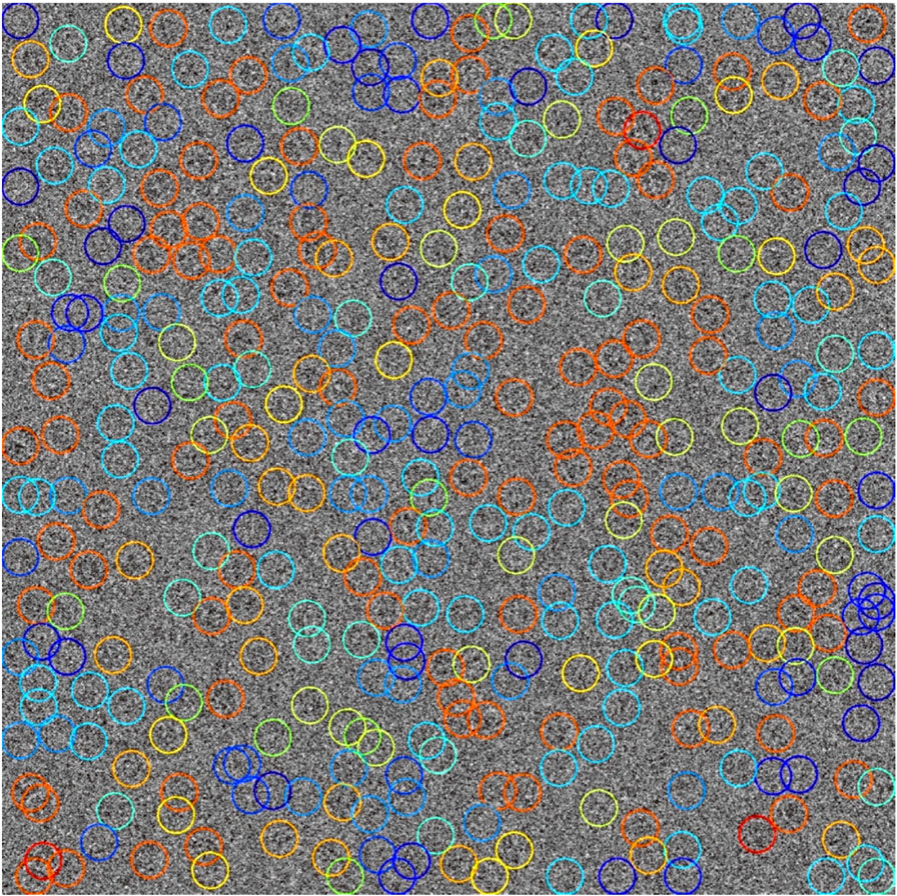
The converged result of the grid-based, local-maximum method of the micrograph from dataset TRPV1 (EMPIAR-10005) [26]. The different colors indicate different levels of particle scores using the same color bar as Fig. 7

The points gradually converge to local maxima during the iterations. Figure 8 shows final results of this micrograph. As the signal-to-noise ratio is too low, the original image is too noisy to be recognized by human. A dark channel haze removal [30] is applied to make the image more readable. The different colors indicate different levels of particle scores using the same color bar as Fig. 7. From this figure, we can see that our method detects most of the particles.

### Experiments on simulated data

We first tested the performance of our method using simulated data generated by InSilicoTEM from PDB-1F07 [24]. As the simulated data contains the ground truth, we can perform detailed experiments to test the accuracy of our method.

**Fig. 9:**
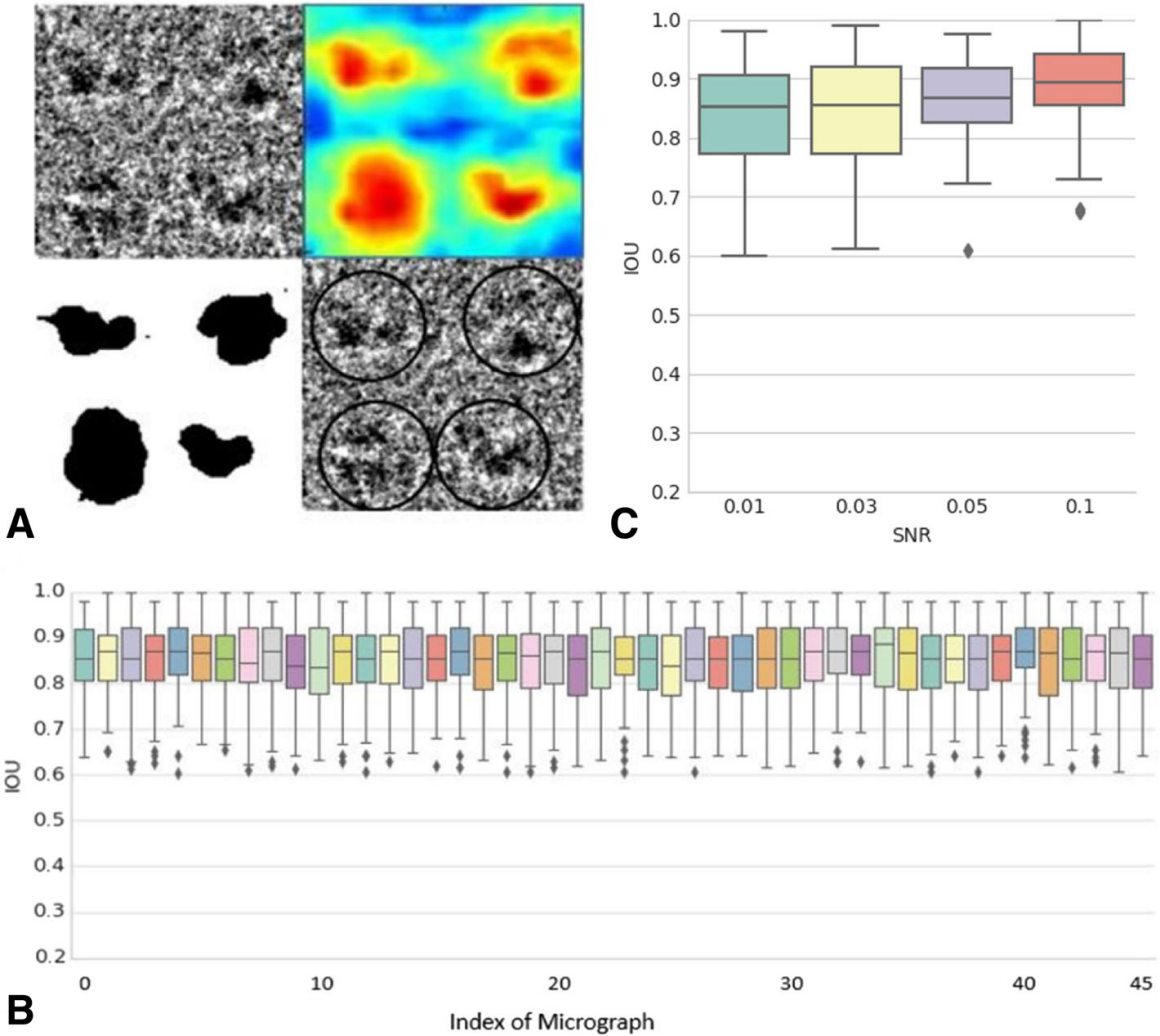
Experiments on simulated data. (**a**) Example of micrographs including the original micrograph, heat map of probability, binarized segmentation results and final coordinates. (**b**) Detailed IOU results of 45 micrographs. (**c**) The IOU results of our method on the simulated data with different SNRs. Here the SNR is defined as $SNR=10{\mathit{\log}}_{10}\left(\frac{\sum \limits_{x=0}^N\sum \limits_{y=0}^M\widehat{f}{\left(x,y\right)}^2}{\sum \limits_{x=0}^N\sum \limits_{y=0}^M{\left[f\left(x,y\right)-\widehat{f}\left(x,y\right)\right]}^2}\right)$, where $\widehat{f}\left(x,y\right)$ is the signal of simulated data generated from InSilicoTEM with no noise, and *f*(*x*, *y*) is the simulated data with noise

Fig. 9a shows one example of the results of the simulated data. In Fig. 9a, the upper left panel is a region of one micrograph. The upper right and lower left panels show the corresponding heat map and binarized segmentation results. The final coordinates are marked in the lower right panel. The final results for this example show that the particle locations are precise. The heat map and binarized segmentation results show that the particles are separated from the background. As the simulated data include the precise location and segmentation results of each particle, we use the pixel IOU to measure performance [27]. We calculated the IOU value for each particle and recorded the statistical information for 45 micrographs (shown in the box plot in Fig. 9b)

Furthermore, as the performance of particle selection methods may vary with different SNRs, we tested our method on the simulated data with different SNRs. Here the SNR is defined as $SNR=10{\mathit{\log}}_{10}\left(\frac{\sum \limits_{x=0}^N\sum \limits_{y=0}^M\widehat{f}{\left(x,y\right)}^2}{\sum \limits_{x=0}^N\sum \limits_{y=0}^M{\left[f\left(x,y\right)-\widehat{f}\left(x,y\right)\right]}^2}\right)$, where $\widehat{f}\left(x,y\right)$ is the signal of simulated data generated from InSilicoTEM with no noise, and *f*(*x*, *y*) is the simulated data with noise. Figure 9c shows the IOU results of our method on different SNRs. As depicted by the figure, IOU drops as SNR decreases. However, even for data with an SNR as low as 0.01, the mean IOU of our method can still achieve 0.86. This result shows the robustness to noise of our method.

### Experiments on real-world data

Our method performed well on simulated data. However, simulated data is simpler than the real-world datasets. To show the robustness and practicality of our method, we performed particle selection on one popular benchmark KLH [28] (Keyhole Limpet Hemocyanin) and three real-world datasets: bacteriophage MS2 (EMPIAR-10075) [25], TRPV1 (EMPIAR-10005) [26] and rabbit muscle aldolase [29] (EMPIAR-100184). The detailed information on these four datasets is shown in Table 2. The training dataset is exactly the data in Table 1. No data in Table 2 are involved. Additionally, we compared our method with three mainstream particle-selection methods: RELION, DeepEM and DeepPicker.

**Table 2:** Data used in the test datasets

Name	10,075	10,005	KLH	10,184
Number of Images	184	100	82	120
Particle Size	300*300	180*180	272*272	256*256
Size of Micrograph	4096*4096	3710*3710	2048*2048	3838*3710
Pixel Size	1.14	1.22	2.2	0.85

**Fig. 10:**
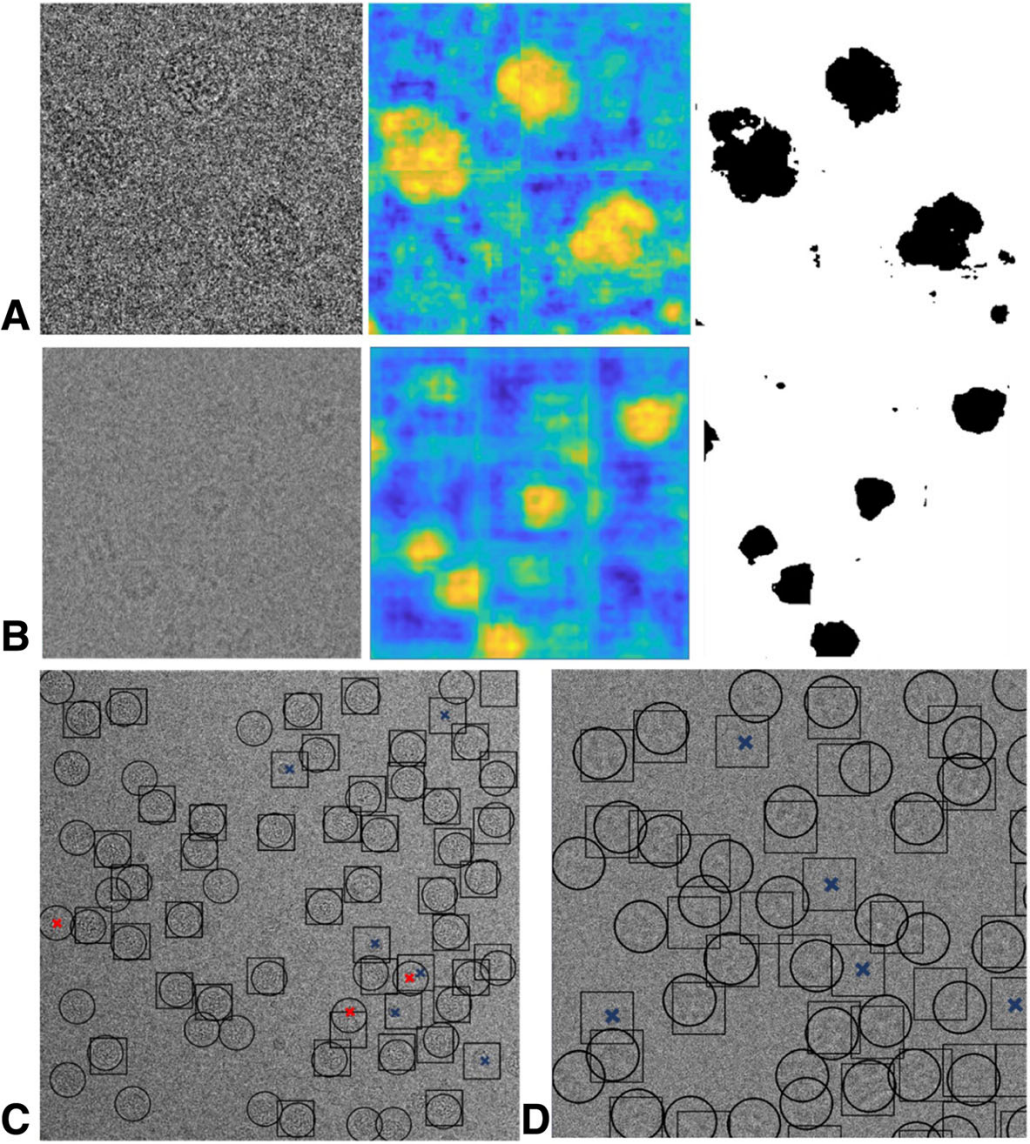
Examples of results for the bacteriophage MS2 and TRPV1. (**a**) Probability density map and the corresponding binarized segmentation results of bacteriophage MS2. (**b**) Probability density map and the corresponding binarized segmentation results of TRPV1. (**c**) Example of particle-selection results from the PIXER and RELION methods on bacteriophage MS2. Circles and rectangles indicate results from PIXER and RELION, respectively. The red and blue crosses in Fig. 10c show the FP particles for PIXER and RELION, respectively. (D) Example of the particle-selection results from the DeepPicker and PIXER methods on TRPV1. We use circles and rectangles to denote results from PIXER and DeepPicker, respectively. We also used blue crosses to indicate the FP results of DeepPicker

To show the quality of the results intuitively, we used dataset bacteriophage MS2 (EMPIAR-10075) and dataset TRPV1 (EMPIAR-10005) to demonstrate the results. We first show examples of the probability density map and the corresponding binarized segmentation results of bacteriophage MS2 and TRPV1 in Fig. 10a and Fig. 10b. As the sizes of micrograph images are too large (4096*4096 for TRPV1), there is not enough memory on the Tesla K20c to generate their segmentation results. Hence, we cropped images into 1024*1024 sub-images. It should be noted that the subtle horizontal and vertical line shown in the density map in Fig. 10a are by-products of this operation. As shown, the influence of the margin is so small that it does not interfere with the particle location. By default, we do not resize the input micrograph to ensure the accuracy of segmentation results. While, we offer the option to down-sample the micrograph in our PIXER, so that we can acquire the result without cropping and merging. Experimental results show that, the performance of PIXER doesn’t decrease with the operation of down-sampling.

We choose two representative methods (one semi-automated particle selection method, RELION, and one full-automated particle selection method, DeepPicker) as the comparisons to show the particle selection result. For the dataset bacteriophage MS2 (EMPIAR-10075) dataset, we show the results comparison with RELION. As its method is semiautomated, we selected approximately 200 particles manually to help to generate the template of particles. Then, we compared the results from PIXER with RELION’s results. In this dataset, the SNR for some of the micrographs is quite high. For these micrographs, we found that the performance of both methods is similar. However, for micrographs with lower SNR, such as the one shown in Fig. 10c, our method detects more particles. We use circles and rectangles to denote the results from PIXER and RELION, respectively. The red and blue crosses in Fig. 10c show the FP particles for PIXER and RELION, respectively. For the dataset TRPV1, its SNR is very low and some of the micrographs are affected by ice contamination. We compared our method with another fully automated deep-learning-based particle-selection method, DeepPicker. To ensure a fair comparison, we used the native model of DeepPicker to perform the experiments. The training data for this model include 10,000 TRPV1 particles. For our fully automated method, there is no intersection between the training dataset and the test dataset (TRPV1); thus, overfitting can be prevented and a more precise evaluation of the performance can be guaranteed. We used the data from Table 1 as the training dataset. No particles or micrographs from TRPV1 reside in our training dataset.

In Fig. 10d, we use circles and rectangles to denote results from PIXER and DeepPicker, respectively. We also used blue crosses to indicate the FP results of DeepPicker. As shown, our method detected more particles with fewer FP results.

**Fig. 11:**
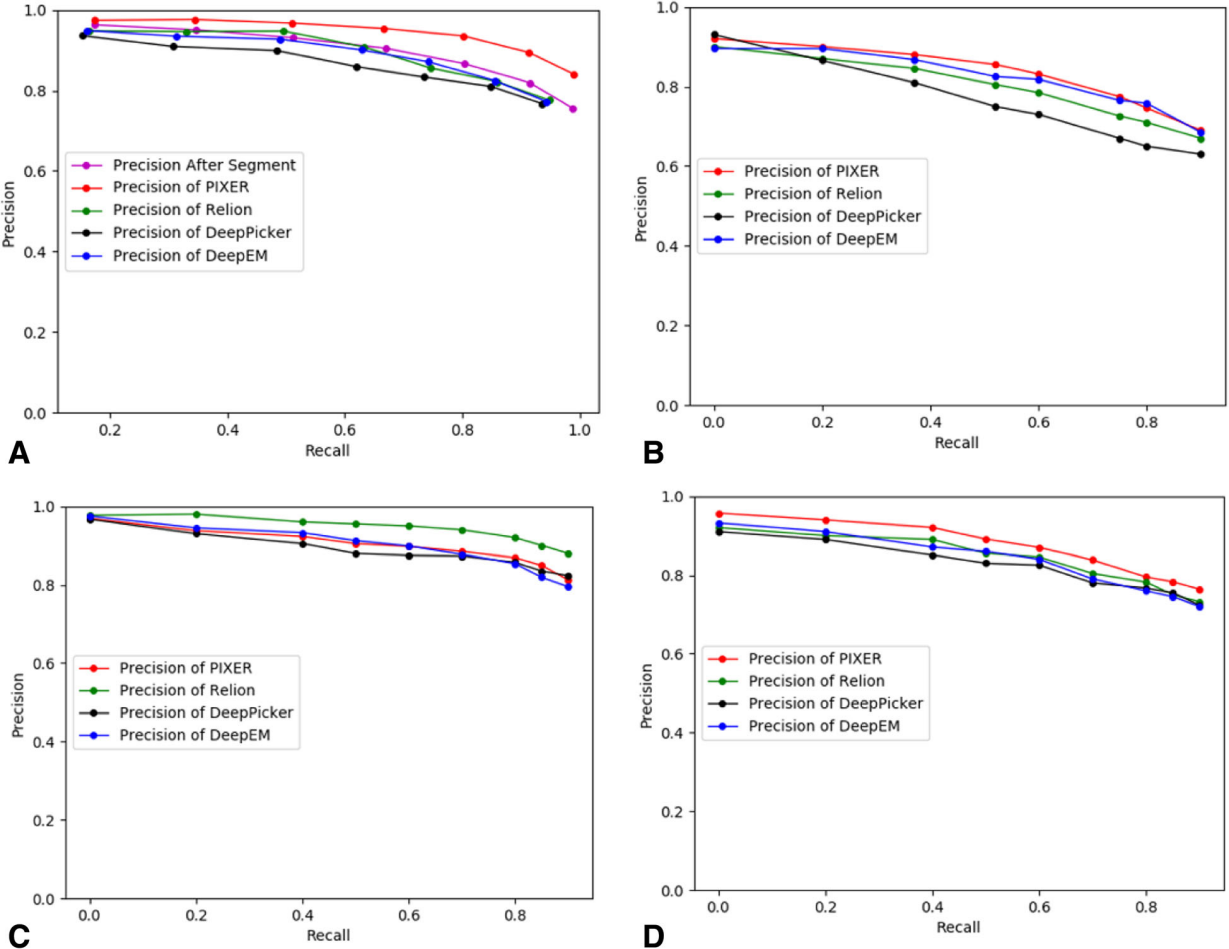
Quantity analysis on real datasets using a precision-recall curve. (**a**) Bacteriophage MS2. (“Precision After Segment” indicates the preliminary results outputted by the segmentation network of PIXER, which haven’t been filtered by classification network.) (**b**) TRPV1. (**c**) KLH. (**d**) Rabbit muscle aldolase

To provide a quantitative analysis of the performance of our method, we compared our method with two mainstream semi-automated particle selection methods (RELION and DeepEM) and one full-automated method (DeepPicker). For the DeepEM method, we used 200 positive or negative images for each dataset as the training dataset to train their own network. We used the manually selected results from experts as the ground truth and recorded the number of true-positive (TP) and false-positive (FP) particles. Here, we used precision ($\mathrm{precision}=\frac{TP}{TP+ FP}$) and recall ($recall=\frac{TP}{TP+ FN}$) to measure the performances of the four datasets (bacteriophage MS2: Fig. 11a),TRPV1: Fig. 11b, KLH: Fig. 11c and rabbit muscle aldolase: Fig. 11d).

In these experiments, there are some parameters need to be set: 1) Particle size. We set the particle size according to Table 2. 2) Lower bound for classification network is set as 0.6 by default. This hyper-parameter is used to distinguish non-particles from particles according to the output of classification network. 3)Maximum selected number of particles per micrograph. In these experiments, to test the ability of removing FP particles for classification network, we leave this parameter as default value 500. In practice experiments, this parameter can help remove the FP particles.

For dataset bacteriophage MS2 (EMPIAR-10075), two different results are shown for our method in Fig. 11a: one is generated from the grid-based, local-maximum selection method without verification from the classification network; the other result is derived from the entire PIXER procedure. As the classification network removes the effects of ice contamination and background noise, the precision is greatly improved. Generally, we find that there are no methods that can always achieve the best performance in different datasets. For the benchmark KLH (Fig. 11c), we find that the template-based method RELION out-performed the deep learning methods. However, for the datasets bacteriophage MS2 (Fig. 11a) and rabbit muscle aldolase (Fig. 11d), our method reached the highest performance. For dataset TRPV1 (Fig. 11b), our method performed as well as DeepEM.

### Computational efficiency

The network is implemented based on ‘Deeplab’ [14], which is a modified version of Caffe. In Deeplab, an Atrous convolution layer is added to enhance the capacity to process multiple-scale objects. In addition, we speed up the pre- and post-processing part of PIXER using MPI and GPU with Python. In our experiment, 6 MPI processes were used in both pre- and post-processing. The source code can be found at GITHUB (https://github.com/ZhangJingrong/PIXER). We set up a GeForce K20c GPU with CUDA 8.0 to train the model and to run the test process of PIXER.

To show the time efficiency, the average time cost of each sub-step (preprocessing, test in the segmentation network, test in classification network and postprocessing) was recorded in Table 3. As can be seen, generally, the processing time increases with the size of the micrograph. For one micrograph with a size smaller or equal to 4096*4096, we can obtain the results within 1 min. We also compared the time performance with the other deep learning based method: DeepPicker [9] and DeepEM [10]. In the last two rows of Table 3, we also show the comparison of their running times. As can be seen, these three methods can process one micrograph in minutes. However, the processing time we need is less than DeepPicker and comparable with DeepEM.

**Table 3:** The time cost of each part of PIXER (Unit: s)

	Pdb1f07	KLH	10,005	10,184	10,075
Micrograph Size	1024*1024	2048*2048	3710*3710	3838*3710	4096*4096
Particle Size	100*100	272*272	180*180	256*256	300*300
Preprocessing	0.17	0.57	3.08	3.72	3.75
Segmentation	0.55	3.64	9.92	9.58	11.23
Classification	2.26	3.12	8.63	6.84	2.75
Postprocessing	0.34	4.59	20.03	6.99	30.38
Total Time	3.32	11.94	41.67	47.17	48.07
DeepPicker	10.47	23.75	80.76	81.34	95.43
DeepEM	40.56	80.54	65.47	39.75	54.38

## Conclusion

In this work, we established an automated particle-selection method (PIXER) based on a segmentation network. First, we use the novel approach of applying a segmentation network to solve the particle-selection problem. Our network can accommodate multiscale particles and micrograph of varying sizes without using a sliding window. Second, facing the challenges associated with assembling training data, we developed a method to generate training data for segmentation. Third, we developed a grid-based, local-maximum selection method to detect particles according to the density map. The results indicated that, as a fully automated method, PIXER can acquire results as good as those achieved using semi-automated methods. However, the potential of the probability density map needs to be further explored. Furthermore, we have not yet introduced a dynamic updating strategy for our method, and this will be the focus of future work.

## Abbreviations

2D: Two-dimensional
3D: Three-dimensional
Cryo-EM: Cryo-electron microscopy
SNR: Signal-to-noise ratio
